# 中国血友病B腺相关病毒载体基因治疗临床应用指导意见（2025年版）

**DOI:** 10.3760/cma.j.cn121090-20250304-00110

**Published:** 2025-05

**Authors:** 中华医学会血液学分会血栓与止血学组 中国血友病协作组

## Abstract

血友病B的主要治疗方式是凝血因子Ⅸ或非因子药物治疗，但是由于无法治愈该病，患者需要终身治疗。近年来血友病B基因治疗得到了长足的发展，国内外共有3种腺相关病毒（AAV）载体血友病B基因治疗产品获得上市许可，其中波哌达可基在中国新近获批。AAV载体基因治疗有着治疗后不可逆以及获得潜在长期疗效的特点，但是在前期临床试验中也有疗效不佳或者疗效丢失的病例。是否适合接受AAV载体基因治疗主要基于患者诊断分型、年龄、抑制物状态、AAV衣壳抗体滴度以及患者/家属意愿等因素。鉴于AAV载体血友病基因治疗已经成为患者可及的前沿治疗，中华医学会血液学分会血栓与止血学组联合中国血友病协作组共同制定本指导意见，通过规范合理的操作及随访建议，使患者在接受基因治疗这种新治疗手段时得到规范处理。

## Abstract (en)

The mainstay of treatment for hemophilia B is Factor Ⅸ or non-factor drug therapy; however, as the disease is incurable, lifelong treatment is required. In recent years, gene therapy for hemophilia B has advanced significantly, with three adeno-associated virus（AAV）vector-based gene therapy products receiving market authorization globally. Among these, Palbociclib was recently approved in China. AAV vector gene therapy is characterized by its irreversible post-treatment effects and potential for long-term efficacy; however, suboptimal efficacy or loss of efficacy has been observed in some cases during early clinical trials. Eligibility for AAV vector gene therapy primarily depends on several factors, including patient diagnostic subtype, age, inhibitor status, AAV capsid antibody titer, and the preferences of the patient and/or their family. Given that AAV vector-based gene therapy for hemophilia has become an accessible frontier treatment, the Thrombosis and Hemostasis Group of the Chinese Society of Hematology, Chinese Medical Association, and the Hemophilia Treatment Center Collaborative Network of China have jointly formulated this guidance. This guidance aims to standardize operational procedures and follow-up recommendations, ensuring that patients receive standardized management when undergoing this novel therapeutic approach.

血友病B是一种Ⅹ染色体连锁隐性遗传性出血性疾病，其发病机制是F9基因突变引起凝血因子Ⅸ（FⅨ）含量下降或者活性降低，临床表现为自发性或外伤后过度出血，严重时危及生命。重型患者典型表现为反复自发性肌肉或关节出血，终将会导致残疾。世界血友病联盟（WFH）在2022年发布的报告显示全球男性中血友病B的患病率为3.8/10万。在中国，2007年至2021年期间全国血友病登记系统共登记3 782例血友病B[1]。目前国内血友病B患者主要接受凝血因子替代治疗，可及药物包括血源FⅨ、重组FⅨ及凝血酶原复合物（PCC），均需要通过静脉输注给药。由于替代治疗无法治愈血友病B，因此患者面临终身频繁静脉穿刺。近些年来，基因治疗得到了迅速发展，国内外共有三种以腺相关病毒（AAV）为载体的血友病B基因治疗药物获批上市或完成Ⅲ期临床试验，为血友病B功能性治愈带来希望。鉴于基因治疗成为中国血友病B患者可以选择的一种前沿疗法，中华医学会血液学分会血栓与止血学组联合中国血友病协作组共同制定本指导意见，以期为中国血友病B AAV载体基因治疗（以下文中简称“血友病B基因治疗”）的临床应用提供理论依据和技术参考，通过规范合理的操作及随访建议，使患者在接受基因治疗这种新治疗手段时得到规范处理。

一、血友病B基因治疗原理及现状

基因治疗包括引入正常功能基因、编辑突变基因以治疗疾病或使基因失活以减少伤害[2]。基因治疗的独特之处在于其治疗的不可逆及长期疗效，血友病B基因治疗的目标是通过一次治疗获得长期甚至终身的减少患者出血倾向的疗效，可称之为临床治愈。目前血友病B基因治疗的策略主要为通过肝脏靶向性高的重组腺相关病毒（rAAV）将高活性FⅨ突变体如FⅨ-Padua（R338L）基因传递到患者肝脏细胞，使其恢复合成具有凝血功能的FⅨ以达到治疗疾病的目的[3]–[4]。AAV载体的优势在于其非致病性，此外，AAV引发的先天免疫反应较弱且持续时间短，临床影响极小[5]。AAV经过基因工程改造，用目的基因取代其原有的病毒序列，从而形成rAAV载体。

目前国内外共有3种血友病B基因治疗药物完成Ⅲ期临床试验，其中波哌达可基（Palbociclib）是由中国公司自主研发的血友病B基因治疗药物。表1列举了3种产品的信息。虽然不同产品在剂量、免疫抑制治疗方案及干预时机上有所不同，但载体衍生因子Ⅸ活性（FⅨ∶C）水平均存在剂量依赖性。此外，载体生产平台、宿主免疫反应（AAV衣壳中和性抗体及针对肝细胞的细胞免疫反应等）以及其他尚不明确原因均可能对最终疗效有一定影响。

**表1:** 国内外完成Ⅲ期临床研究的血友病B基因治疗药物[6]–[12]

通用名/代码	Etranacogene Dezaparvovec	Fidanacogene Elaparvovec	波哌达可基
剂量（vg/kg）	2×10^13^	5×10^11^	5×10^12^
载体衣壳	AAV5	AAV-Rh74var	AAV843
携带基因	ss-CO-FⅨ-R338L	ss-CO-FⅨ-R338L	ds-CO-FⅨ-R338L
AAV抗体要求	未纳入入排标准	中和抗体滴度≤1∶1	中和抗体滴度<1∶4，结合抗体滴度≤1∶200
产品审批状态（截至2025年5月）	欧盟/美国/加拿大获批	欧盟/美国/加拿大获批	中国获批
治疗后平均FⅨ∶C	12个月（41.5±21.7）％，24个月（36.7±19.0）％	15个月（26.9±25.1）％，24个月（26.6±24.9）％	12个月（55.08±35.93）％

**注** AAV：腺相关病毒；ss：单链；ds：双链；CO：密码子优化。FⅨ∶C：凝血因子Ⅸ活性，检测方法为基于SynthaSIL aPTT试剂的一期法

二、开展基因治疗医疗机构的要求和建议

作为一种创新性治疗方式，目前尚无针对开展基因治疗医疗机构资质认定的行业标准。通过中国血友病中心建设评估的血友病中心，尤其是血友病综合管理中心及血友病诊疗中心，具备基因治疗所需要的血友病诊断、持续监测及多学科合作能力，但各家中心对于血友病基因治疗的经验不同。为了保证药品正确配置、给药及安全，医疗机构需要配备生物安全柜、静脉输液泵、监护设备、急救/抢救设备等常规硬件。为了对患者的疗效（FⅨ∶C、关节健康评估等）、特别关注事件（肝脏转氨酶）及其他安全性指标进行监测，建议成立由血液科、康复科、药剂科、肝病/消化科、检验科等相关专业医师及血友病护士组成的多学科合作团队，共同参与基因治疗的患者全流程管理[13]–[14]。对于缺乏基因治疗经验的医疗机构，建议于有基因治疗经验的医疗机构学习交流，以规范地开展基因治疗。

三、患者的选择及决策

基因治疗的决策是在医患充分沟通前提下共同决策的过程，在作出最终决定前需要经过以下过程，在接受基因治疗前需要获取知情同意书。

1. 医患沟通基因治疗的时机：医师可以主动，或者在患者表达基因治疗意愿后，通过评估患者信息初步判断患者接受基因治疗的可行性。初步评估内容包含以下方面：

（1）疾病分型及临床表型：重型（FⅨ∶C<1％）或出血频繁的中间型（FⅨ∶C 1％～5％）血友病B患者是基因治疗潜在的获益群体。虽然目前临床试验纳入的均为FⅨ∶C ≤ 2％的血友病B患者，但对于活性虽然较高但仍有频繁出血，或者出现过自发性致死性出血需要长期预防治疗的患者，也可能从基因治疗中获益。

（2）年龄：目前血友病B基因治疗药物已发表临床试验数据和获批适应证中，仅包含≥18岁的成年血友病B患者。

（3）FⅨ抑制物及暴露日：FⅨ抑制物产生常伴有过敏反应甚至肾病综合征，而基因治疗特点之一是不可逆性，因此需要在基因治疗前评估抑制物风险。既往无抑制物阳性史、FⅨ暴露日≥100 d，基因治疗前抑制物阴性患者可以接受基因治疗。

对于进行基因治疗评估时抑制物阴性，但是既往抑制物阳性的患者，目前无基因治疗有效性及安全性数据，建议综合评估患者是否接受过免疫耐受诱导治疗及疗效、FⅨ过敏史、是否出现过肾病综合征、抑制物转阴后FⅨ暴露日天数、是否有抑制物复发史等因素，并与患者充分沟通潜在风险后审慎决定。

2. 充分知情条件下的医患共同决策：在决定基因治疗决策前，需要向潜在患者沟通但不限于以下内容：

（1）基因治疗的原理及不可逆性。

（2）潜在获益及治疗反应：包括现有临床试验及真实世界研究展现的治疗疗效及个体差异性、无效/疗效丧失的风险、无法进行第二次同产品基因治疗、由于AAV衣壳抗体交叉反应性可能无法接受其他AAV载体基因治疗、FⅨ∶C水平稳定在不同范围时血友病的治疗方案等。

（3）伴随治疗及安全性信息：不同血友病B基因治疗药物伴随不同免疫抑制治疗（IST）方案：开始IST的时机、方案、疗程以及重复使用IST治疗的可能（例如在免疫反应导致的转氨酶增高及FⅨ∶C下降时）；生育注意事项；长期安全性等信息。

（4）回答患者关于基因治疗的问题。

四、基因治疗前评估

在基因治疗前，需要进行一系列的评估，以判断其潜在的获益及安全性，除上述提及的临床指标外，还需要评估以下方面:

1. 预存AAV抗体检测：基于基因治疗药物所使用AAV亚型和临床试验数据，需满足以下要求方可应用对应药物：（1）Fidanacogene Elaparvovec：要求接收治疗患者无针对Rh74var亚型的AAV中和抗体；（2）Etranacogene Dezaparvovec：原则上对AAV抗体检测结果无要求，但鼓励患者在治疗前进行AAV5抗体检测（对于抗体滴度高于1∶678的患者，需格外关注）[15]；（3）波哌达可基：AAV 843中和抗体滴度≤1∶4，结合抗体滴度≤1∶200。

对于超过上述抗体滴度要求的患者，基因治疗疗效并未得到临床试验的验证，应谨慎选择。

2. 肝脏健康评估：表2中列举的可接受肝功能标准来自不同产品Ⅲ期临床试验中所采纳的标准。

**表2:** 临床研究中不同血友病B基因治疗药物对肝功能相关指标的要求[6]–[12]

肝功能指标	Fidanacogene Elaparvovec	Etranacogene Dezaparvovec	波哌达可基
转氨酶（ALT/AST）	≤2倍正常上限	≤2倍正常上限	≤1.5倍正常上限
碱性磷酸酶	≤2倍正常上限	≤2倍正常上限	无要求
总胆红素	≤1.5倍正常上限	≤2倍正常上限	≤1.5倍正常上限

目前3种血友病B基因治疗药物均未在超出表2中转氨酶标准的患者中进行有效性及安全性研究。但是鉴于3种基因治疗药物的原理，且部分入组患者出现伴随无症状转氨酶增高的FⅨ∶C下降，不建议伴有严重肝病的患者接受基因治疗。包括但不限于以下疾病状况：既往诊断为门静脉高压、脾大、肝性脑病或肝纤维化≥Ⅲ期；B超发现有肝脏结节和囊肿，或实验室检查提示甲胎蛋白升高等，经判断这些异常情况有临床意义的患者不适合此治疗。

3. 其他评估：除以上需要评估指标外，在基因治疗前需要进行详细的临床信息采集及实验室检查。

（1）病史收集及体格检查：收集患者血友病相关病史，包括诊断日期、严重程度、出血史、关节病变情况、既往治疗情况等。此外，需要重点收集患者既往是否有肝脏及其他感染性疾病史、饮酒史、过敏史、婚育史及生育计划等信息。体格检查项目包括：身高、体重信息，出血频率，靶关节数目及位置，关节健康（包括双侧股骨头健康状态），血栓风险及生活质量评估等。

（2）相关实验室及影像学检查：患者接受基因治疗前需完成以下实验室检查：血、尿、便常规，肝、肾功能，FⅨ∶C，FⅨ抑制物检测，乙肝病毒、丙肝病毒及HIV相关病毒学检查，甲胎蛋白等。接受基因治疗前3个月内需完成AAV抗体滴度检测。

此外需完成肝脏相关影像学检查（超声、CT或者MRI等）评估肝脏健康状态，完成关节超声或其他影像学检查评估靶关节病变。

五、基因治疗药物应用及伴随药物

（一）预防性免疫抑制剂的应用

机体针对AAV转导肝细胞的细胞免疫可能会导致转氨酶升高，FⅨ表达下降等情况[16]–[17]，使用免疫抑制剂可以降低免疫反应，防止肝细胞损伤及转氨酶增高。不同基因治疗药物在临床试验过程中采用了不同的IST方案，为上市后的应用提供了参考。但是目前并没有明确、统一的的IST方案，医疗机构需要根据患者相关并发症、基础肝功能水平、不同免疫抑制剂耐受程度，以及结合临床试验阶段的IST方案，制定相应方案。不同产品在临床试验阶段IST方案如下[6]–[12]：

Fidanacogene Elaparvovec：筛选后至用药前，患者ALT/AST单次升高≥1.5倍基线，无论是否超过正常范围，可考虑使用免疫抑制剂。以泼尼松为例，推荐起始剂量60～100 mg/d。

Etranacogene Dezaparvovec：用药前不常规使用免疫抑制剂。

波哌达可基：建议所有患者在用药前1 d开始应用免疫抑制剂。体重≤80 kg者，泼尼松起始剂量为1 mg·kg^−1^·d^−1^；体重>80 kg者，起始剂量为80 mg/d。用药1周后开始逐步减量，如未发生转氨酶增高，一般8～10周左右减停。

临床应用之前，建议筛查患者是否存在高血压、消化性溃疡、精神疾病、青光眼、股骨头坏死等可能不适合使用糖皮质激素的情况。如患者存在糖皮质激素应用禁忌证，可考虑使用其他替代性免疫抑制剂（如使用他克莫司，需注意监测血药浓度）。

（二）基因治疗药物运输、保存及稀释

Fidanacogene Elaparvovec需要在−100 °C～−60 °C环境下运输及保存，配制后应保存于2 °C～8 °C，24 h内输注。Etranacogene Dezaparvovec需要在2 °C～8 °C环境下运输及保存，配制后应避光保存于15 °C～25 °C，24 h内输注。波哌达可基需要在−90 °C～−70 °C环境下运输及保存，配制后应保存于10 °C～30 °C，8 h内输注。

（三）基因治疗药物注射

基因治疗后载体衍生FⅨ∶C提高的时间在不同药物中各不相同，在FⅨ∶C尚未提高至有效水平时可以继续使用外源性FⅨ制剂替代治疗，具体可参照各产品说明书。波哌达可基在临床试验中发现载体输注第2天即可检测出FⅨ表达，并快速增加，在1周时即可接近最高水平，可使患者快速脱离自发性出血风险。

基因治疗药物应于低温保存及运输，输注当日取出，置于室温自然融化。根据受试者体重，计算不同药物所需用量：Fidanacogene Elaparvovec 5×10^11^ vector genome/kg（vg/kg），Etranacogene Dezaparvovec 2×10^13^ vg/kg，波哌达可基 5×10^12^ vg/kg。建议在生物安全柜内采用无菌操作技术进行配制，配置后需要在规定时间内输注完毕。

提前建立静脉通路，用合适大小和数量的注射器抽取所需体积的药物，然后使用注射泵以规定速度完成一次性静脉输注。输注后，使用适量0.9％氯化钠溶液冲洗管路，确保残留在管路中的药物全部输注至患者体内。

基因治疗药物输注时可能发生输液相关反应，包括寒战、发热、皮疹、头晕、头痛、胸闷、腹痛、呼吸困难、喘息、晕厥、血压异常（低血压或高血压）等。在整个输注期间以及输注结束后至少3 h内密切监测患者输液相关反应的体征或症状。在给药过程中如发生输液相关反应时，可减缓输注，或暂停输注直至症状缓解。重新开始输注时，应以较慢的速度重新开始。可使用糖皮质激素或抗组胺药等对症处理。

六、基因治疗后随访评估

（一）有效性评估

1. FⅨ∶C监测及临床疗效评估：患者接受基因治疗后有效性评估应包含FⅨ∶C及临床疗效两个方面。基因治疗后FⅨ∶C监测频率建议参考表3。如患者出现因子水平下降趋势，可增加监测频率。临床疗效评估包括患者出血频率、靶关节出血情况、关节查体及影像学评估、生活质量评分等。

**表3:** 推荐基因治疗后凝血因子Ⅸ活性及肝脏转氨酶监测频率

基因治疗后时间	监测频率
输注后第1周内	每周2次（输注后第3天、第6天）
输注后第2～12周	每周1次
输注后第13～26周	每2周1次
输注后第27～52周	每4周1次
输注后第1～2年	每3个月1次
输注后第2～5年	每6个月1次
输注后5年以上	每年1次或2次

需要注意的是，在使用基于活化部分凝血活酶时间（aPTT）的一期法检测FⅨ∶C时，不同aPTT试剂的检测结果有所差异，例如使用SynthASil（Instrumentation Laboratory公司, 美国）检测的FⅨ∶C是ACTIN FSL（Siemens Healthineers, 德国）检测活性的1.35倍左右，因此在解读患者FⅨ∶C水平时需要注意检测试剂的差异[18]。同一例患者在随访过程中建议使用相同检测方法和相同试剂进行FⅨ∶C监测。

随访过程中患者可能出现伴随转氨酶增高的FⅨ∶C下降或不明原因FⅨ∶C下降，此时是否需要启动免疫抑制剂治疗，可参考以下标准[6]–[12]：

（1）Fidanacogene Elaparvovec：连续两个时间点检测发现ALT/AST持续升高，无论是否超过正常范围；FⅨ∶C水平下降：可能会导致出血发生的不明原因FⅨ∶C下降或连续两个时间点监测均有下降（尤其在治疗后的前4个月）。以泼尼松为例，起始剂量为60～100 mg/d，在转氨酶恢复正常和（或）FⅨ∶C下降趋势趋于稳定后逐步减量。

（2）Etranacogene Dezaparvovec：在基因治疗后前3个月每周检测ALT及AST水平，如果转氨酶增高，应持续监测肝功能直至其恢复至基线值。如果ALT高于正常值上限或基线2倍以上，考虑开始糖皮质激素治疗，推荐起始剂量为泼尼松60 mg/d，ALT恢复后逐渐减量。

（3）波哌达可基：在基因治疗后如果出现ALT和（或）AST>1.5倍基线或高于正常值上限、非FⅨ抑制物原因持续出现FⅨ∶C下降时，可以启动IST，泼尼松起始剂量为1 mg·kg^−1^·d^−1^，体重>80 kg者使用泼尼松80 mg/d起始剂量。如果患者在预防性泼尼松使用过程中出现转氨酶增高，可以恢复至初始剂量。

2. 基因治疗后替代治疗方案：虽然不同aPTT试剂方法检测FⅨ∶C存在差异，但哪种检测方法能够反映真实凝血能力，尚无定论。基因治疗后应根据FⅨ∶C及临床表型制定个体化治疗方案。

（1）接受基因治疗后患者FⅨ∶C<1％或较基线值无明显增加，视为无效；有效患者FⅨ∶C降至基线水平视为疗效丧失。以上患者可能需要恢复既往治疗。

（2）转变为中间型血友病：基因治疗后FⅨ∶C 1％～5％且高于基线值，尤其是FⅨ∶C达到3％或更高时，可尝试停止预防性治疗或减少预防性治疗的频率，转为按需治疗。根据患者的出血表型（尤其是FⅨ∶C<2％患者），可能仍需要预防治疗。

（3）转变为轻型血友病：FⅨ∶C≥5％时，可以停止预防治疗，仅在出血和手术时按需使用凝血因子进行治疗。

（4）反应良好至极佳：FⅨ∶C>15％～20％，此时可能仅在重大手术时才需要替代治疗。

（5）血友病B患者基因治疗后的围手术期处理：需要结合患者FⅨ∶C以及具体手术方式而定。《新英格兰医学期刊》2022年刊登了首例血友病B患者基因治疗后接受全膝关节置换手术而无需额外FⅨ替代治疗的病例[19]。该患者术前FⅨ∶C约为50％（一期法，Actin FSL aPTT试剂），手术当日短暂降至40％～50％后恢复至50％以上。该病例表明基于Actin FSL的一期法检测FⅨ∶C 50％时可以在大手术中提供足够的凝血能力，但是其他aPTT试剂检测FⅨ∶C 50％时是否可以耐受大手术，尚不明确。整体而言，如果在无替代治疗保护下进行手术时发生了出血，应及时给予FⅨ制剂替代治疗。

（二）安全性评估

1. 肝脏健康状态的随访及处理：肝细胞是合成FⅨ的关键部位，也是AAV介导血友病B基因治疗的靶细胞[20]。患者接受基因治疗后出现的不明原因的无症状转氨酶（ALT及AST）升高可被视为肝脏内细胞免疫反应的生物标志物，提示炎症反应的存在，甚至可能对转基因的表达下降有一定的预测参考价值。在临床试验中观察到的转氨酶升高多为轻度到中度，多数情况下应用免疫抑制剂治疗有效。因此推荐应用基因治疗后规律监测ALT和AST水平，具体监测频率可参考表3；如果出现转氨酶波动，可酌情增加监测频率。在随访过程中出现转氨酶升高时，要排除其他原因导致的肝功能损伤（如基础肝病、药物等），同时需要关注FⅨ∶C水平，考虑启动IST治疗。必要时，可考虑请消化、肝病科等相关科室会诊。

除此之外，建议每半年至1年复查甲胎蛋白及腹部超声或CT/MRI等肝脏影像学检查。

接受基因治疗的血友病B患者，日常需注意控制体重、避免饮酒、避免可能导致肝毒性的药物及食物、降低脂肪肝、酒精性肝病、药物性肝损伤等发生风险。

2. 肿瘤风险的随访评估：rAAV载体将目的基因递送进入细胞核内，形成游离于基因组的双链环形DNA，仅有微量目的基因可以整合进入宿主基因组中，结合目前尚未有与基因治疗相关的肿瘤形成报道，因此肿瘤风险极小。对于出现肿瘤的患者，可考虑通过肿瘤组织二代测序（NGS）、全基因组测序等手段帮助判断与AAV基因整合的相关性[21]。

3. FⅨ抑制物监测与处理：虽然目前3种血友病B基因治疗药物临床试验中均未见FⅨ抑制物出现的报道，但是建议在实际临床应用中每年检测抑制物1次，或者当患者出现不明原因FⅨ∶C下降或出血频繁时行抑制物检测。一旦确认FⅨ抑制物阳性，可参照《血友病合并抑制物诊断与治疗中国指南（2023年版）》[22]等相关指南处理。

七、对于基因治疗后患者生活方式的建议

血友病B患者接受基因治疗后要保持良好的生活习惯和均衡饮食，摄取足够的蛋白质、维生素、矿物质和其他营养素，以满足正常生理功能需求。需注意避免危害肝脏健康的饮食，如饮酒、长期高热量及过度油腻食物等。

患者接受基因治疗后应在医师指导下根据FⅨ∶C检测结果进行相应强度的活动或运动，避免出血。需要注意的是，损伤严重的关节较健康关节更容易发生出血，因此需要结合患者FⅨ∶C以及关节健康制定活动方案，如发生出血，应及时给予FⅨ制剂止血并谨慎从事该强度活动或运动。

目前未见关于基因治疗对女性或男性生育能力影响的报道。药物药代动力学结果显示，无论接受何种基因治疗药物输注，AAV载体基因会脱落至精液中，并逐渐清除[9]–[10]。因此，建议男性患者在接受基因治疗后有效避孕1年，1年后如有生育计划，应咨询医生。

除此之外，由于患者往往对基因治疗抱有较高期望，面对基因治疗后治疗方式改变、生活方式改变、疗效的个体差异（疗效不佳或疗效丢失）等情形，患者在心理上会产生一定改变。因此有必要在基因治疗的全流程中给予必要的心理支持治疗。心理科医师需要和临床医师一起在治疗前让患者对基因治疗有充分的认知、建立合理的心理预期；治疗后面对疾病身份和心态的调整，生活、工作方式的改变以及随访过程中如果出现相关医学问题如何保持情绪稳定，都需要心理专家积极参与[23]–[25]。

八、总结

血友病基因治疗在全球范围内尚处于起步阶段，较传统凝血因子替代治疗体现出显著临床优势。血友病B基因治疗仍需不断探索和优化，其安全性与有效性需要更多临床试验数据支持。本指导意见可为血友病B基因治疗的临床试验和应用提供参考和依据，更好地促进基因治疗的规范开展。
